# Phthalate Plasticizers in Children’s Products and Estimation of Exposure: Importance of Migration Rate

**DOI:** 10.3390/ijerph17228582

**Published:** 2020-11-19

**Authors:** Du Yung Kim, Sa-Ho Chun, Yerin Jung, Dana Fahad Mohamed Salman Mohamed, Hae-Soo Kim, Da-Young Kang, Jeong-Won An, Seong-Yeol Park, Hyun-Wook Kwon, Jung-Hwan Kwon

**Affiliations:** 1 Division of Environmental Science and Ecological Engineering, Korea University, 145 Anam-ro, Seongbuk-gu, Seoul 02841, Korea; duyungkim@korea.ac.kr (D.Y.K.); qtwree@korea.ac.kr (Y.J.); danasalman@korea.ac.kr (D.F.M.S.M.); 2 FITI Testing & Research Institute, 21 Yangcheong 3-gil, Ochang-eup, Cheongwon-gun, Cheongju-si, Chungcheongbuk-do 28115, Korea; shchun@fiti.re.kr (S.-H.C.); haesoo@fiti.re.kr (H.-S.K.); dykang@fiti.re.kr (D.-Y.K.); jwan@fiti.re.kr (J.-W.A.); Psy065@fiti.re.kr (S.-Y.P.); khw@fiti.re.kr (H.-W.K.)

**Keywords:** children, phthalate esters, plasticizers, consumer products, exposure assessment, migration

## Abstract

Plasticizers are added to diverse consumer products including children’s products. Owing to their potential for endocrine disruption, the use of phthalate plasticizers is restricted in many children’s products. In this study, exposure to five phthalate esters (dibutylphthalate, di(2-ethylhexyl) phthalate (DEHP), diethyl phthalate, di-isobutyl phthalate, and diisononyl phthalate (DINP)) and an alternative (di-ethylhexyl adipate) was assessed by the use of children’s products based on chemical analysis of 3345 products purchased during 2017 and 2019 in Korea. Plasticizers were found above the detection limits in 387 products, and DEHP and DINP were the two most predominantly detected plasticizers. Deterministic and probabilistic estimation of the margin of exposure at a screening level revealed that the use of children’s products might be an important risk factor. However, it is also highly likely that the exposure could be overestimated, because the migration rate was estimated based solely on the content of plasticizers in children’s products. Chemical migration is a key process determining the absorption of plasticizers from products; thus, further refinements in experimental determination or model estimation of the migration rate are required.

## 1. Introduction

Phthalate plasticizers are widely used to enhance the elasticity and durability of numerous plastic products [1,2]. With increasing volumes of plastic products being produced, the amount of phthalate plasticizers being used is increasing as well [3]. Many phthalate plasticizers are suspected to be potential endocrine-disrupting chemicals because of their adverse effects on reproductive development [4,5]. Thus, many countries have regulations concerning the use of phthalate plasticizers in consumer products [6,7,8,9,10,11]. Exposure assessment based on the concentration of phthalate plasticizers in children’s products is crucial, because children are more susceptible to those plasticizers.

In recent decades, phthalate plasticizers in children’s products such as toys, teethers, and backpacks were analyzed in numerous studies [12,13,14,15,16,17,18]. They were found in various polymeric parts of a product, especially polyvinyl chloride (PVC). Of the analyzed products, some had two or more phthalate plasticizers, but most products had one phthalate plasticizer. In the early 2000s, diisononyl phthalate (DINP) was the predominant phthalate plasticizer in PVC toys, of which concentration was up to 44%, and di(2-ethylhexyl)phthalate (DEHP) was the second-most dominant [12,13]. From the late 2000s, DEHP became the most used phthalate ester, accounting for more than 50% of total phthalate production [3,19]. In addition to DINP and DEHP, other common phthalate plasticizers have been used in many plastic products. Because of their abundance in children’s products, quantitative assessment of exposure to phthalate plasticizers should be prioritized.

Exposure models, such as the consumer exposure model (CEM) from the U.S. Environmental Protection Agency (EPA) and ConsExpo from the National Institute for Public Health and the Environment of the Netherlands (RIVM), were developed to estimate the human exposure from diverse consumer products through various exposure pathways. Such user-friendly models include various exposure scenarios consisting of product types, exposure pathways, and exposure factors. Many parameters in those estimation models are also provided by default or calculated using basic physicochemical properties such as vapor pressure. Though estimation models are widely used because of their convenience, inherent uncertainties are inevitable in exposure estimation due to the default exposure factors of the model. In CEM, for example, the estimated exposure through ingesting product debris has been strongly affected by the migration rate in the digestive system. Users can choose one among five migration rate values as input data, and this simple choice might lead to an overestimation of exposure. Thus, identification of critical input parameters causing large uncertainties in the estimation and refining the range of uncertain parameters should be very important toward more reliable estimation.

In this study, the concentrations of five phthalate esters (i.e., dibutyl phthalate (DBP), DEHP, diethyl phthalate (DEP), di-isobutyl phthalate (DIBP), and DINP) and an alternative, di-ethylhexyl adipate (DEHA), were analyzed in 3345 children’s products purchased from 2017 to 2019 in Korea to understand the baseline level of those plasticizers in children’s products. Exposure to phthalates and DEHA from children’s products through ingestion and dermal absorption was estimated with measured concentrations in products. Exposure assessment was conducted relying on the exposure algorithms adopted in CEM by both deterministic calculation and probabilistic estimation using Monte Carlo simulation. Finally, the margin of exposure (MOE) of each chemical was calculated and critical parameters determining estimation uncertainty were identified.

## 2. Materials and Methods

### 2.1. Purchase of Samples

Different children’s products (n = 3345) were purchased from on- and off-line stores between 2017 and 2019. They were categorized into six groups: children’s accessories, mats, shoes, stationeries, toilets, and toys. Detailed items in those six product categories are summarized in Table 1. Only the part made of plastic material was separated from a product and subjected to chemical analysis.

### 2.2. Chemicals

For analytical standard, DBP, DEHP, and DINP were purchased as a mixture from AccuStandard (PLAS-CPSC mixture, 500 μg mL^−1^ each, New Haven, CT, USA). Individual standards of DEP, DEHA, and DIBP were purchased from AccuStandard (ALR-110S, 100 μg/mL and P-233S, 1000 μg/mL) and Sigma-Aldrich (152641, 99.7%, St. Louis, MO, USA), respectively. All organic solvents (methanol, tetrahydrofuran, acrylonitrile, etc.) used in sample preparation were of analytical grade.

### 2.3. Sample Preparation and Instrumentation

#### 2.3.1. Sample Preparation

For sample extraction, the proposed procedure of CPSC-CH-C1001-09.4 [20] and Standard Method for Environmental Hazard [21] were applied. Each plastic part was separated from the product and cut into small pieces less than 2 mm or milled into a powder. For extracting plasticizers, approximately 0.3–1.0 g of the weighed sample was submerged in 10–15 mL of tetrahydrofuran in a glass vial. To allow dissolution, the vial was sonicated for 30 min, followed by additional 2 h sonication in case that the sample was not completely dissolved. Then, 10–15 mL of acrylonitrile or methanol was added to precipitate the polymer. After vigorous shaking, suspended particles that formed were allowed to settle for 5 min. The supernatant was then filtered through a 0.45 μm polytetrafluoroethylene filter and analyzed using gas chromatography mass spectrometry (GC-MS). Triplicate analyses were conducted for samples in which phthalate(s) were determined above the quantification limits in the first analysis.

#### 2.3.2. Chemical Analysis

The analysis of the extracted solution was conducted by GC-MS (Shimadzu GCMS-QP2020, Kyoto, Japan). The temperature of the injector was 280 °C and injection volume was 1 μL. Helium was used as carrier gas at 1.0 mL/min. Fused silica capillary column (DB–5ms, 30 m × 0.25 mm × 0.25 μm) was used for the separation. The oven temperature was programmed from 80 °C (held for 0.5 min), raised to 280 °C at 10 °C min^−1^, and subsequently, raised to 320 °C at 25 °C min^−1^ held for 7 min. The temperature of the ion source was 230 °C for detector. NIST 08 MS Library and MS Search Program v.2.0 (The NIST Mass Spectrometry Data Center, 2008) was used for phthalate identification, and the mass spectrum scan range for qualitative analysis was *m/z* 50–500. Mass-to-charge ratios (*m/z*) 149, 205, 223 (for DBP, DIBP), 149, 167, 279 (for DEHP), 149, 293, 307 (for DINP), 65, 149, 177 (for DEP), 129, 147 (for DEHA) were monitored for quantitative analysis.

Limit of quantitation (LOQ) was calculated as 30.6 mg kg^−1^ (DBP), 33.1 mg kg^−1^ (DIBP), 32.5 mg kg^−1^ (DEHP), 35.6 mg kg^−1^ (DINP), 44.0 mg kg^−1^ (DEP), and 41.0 mg kg^−1^ (DEHA), respectively. However, the LOQ was calculated at the level of about 40 mg kg^−1^ as described above, but in consideration of the safety factor, only children’s products detected over 100 mg kg^−1^ were used for exposure assessment.

### 2.4. Exposure Estimation

#### 2.4.1. Exposure Algorithms through Ingestion and Dermal Absorption

Ingestion of product debris or indoor dust, dermal absorption, and inhalation are three major routes of human exposure to phthalate esters and DEHA. Exposure pathways through ingesting indoor dust and inhalation of gaseous chemicals were excluded, because there might be other important sources determining their concentration in indoor air and dust [22]. The exposure through the ingestion of product debris and dermal absorption was estimated with algorithms provided by CEM [23]. Daily dose by ingestion (*Daily dose_ingestion_*) and dermal absorption (*Daily dose_dermal_*) was expressed as Equations (1) and (2), and parameters used in those equations were estimated using Equations (3)–(7).

(1) $$Daily\;dose_{ingestion}=\frac{MR\times CA\times D_{mouthing}\times ED}{BW\times AT}$$

(2) $$Daily\;dose_{dermal}=\frac{C_{art}\times \frac{SA}{BW}\times l\times FA\times ED}{AT}$$

(3) $$l=\sqrt{2\times D\times Dur/60}$$

(4) $$FA=\frac{3+\chi \left[1-\exp\left(-a\frac{Dur}{t_{lag}}\right)\right]}{3\left(1+\chi \right)}$$

(5) $$\chi =\frac{h\times p_{vap}\times MW}{K_{p}\times S_{w}\times R\times T}$$

(6) $$t_{lag}=\frac{h_{sc}}{6\times 10^{-2.8-0.0056MW}}$$

(7) $$K_{p}=\frac{1}{\left(\frac{1}{K_{lip}+K_{pol}}\right)+\left(\frac{1}{K_{aq}}\right)}$$

Definitions of all parameters in the exposure estimation with their units are listed in Table 2.

#### 2.4.2. Deterministic Estimation

The minimum and the maximum daily dose of each plasticizer in a product category were calculated using Equations (1) and (2). For ingestion exposure, the migration rate of each chemical is decided by concentration in a product. The migration rate was assigned to be 10 mg cm^−2^ h^−1^ when the content of the chemical is over 1% (*w*/*w*), and 0.1 mg cm^−2^ h^−1^ when the content is less than 1% (*w*/*w*). In the case of dermal absorption, the highest and the lowest concentrations of each chemical were used to calculate the exposure range. The values of other exposure parameters in Equation (2) were defaults or calculated from input parameters based on CEM.

#### 2.4.3. Probabilistic Estimation

A probabilistic approach to Equations (1) and (2) was also considered to estimate exposure to each chemical. Exposure throughout all product categories was summed by using Monte Carlo simulation to estimate exposure by incorporating uncertainty of the exposure parameters, such as *ED* [24]. Monte Carlo simulation repeatedly draws random parameter values from the probability density function of each parameter and estimates approximate distributions of the result. Crystal Ball software (Oracle, Redwood City, CA, USA) was used for Monte Carlo simulation in Microsoft Excel (Microsoft Corp, Redmond, WA, USA) spreadsheets. After 10,000 iterations, the median and 95% value of the daily exposure dose were obtained.

Probability density function of *MR* and *C_art_* by product categories was estimated using chemical concentrations including not-detected values as half of the detection limits [25]. For estimating the probability density function of parameters, mean and standard deviation values of *CA*, *ED*, *SA,* and *BW* were referred from the Korean children exposure parameter book [26], and the mean and 95 percentile values of *D* were referred from the CEM guidebook [23]. Different values of *ED* were chosen according to product categories: daily duration of stay-in-home for accessories; stay-out-of-home for shoes; playing mat and toy; study for stationery; personal sanitation for toilet. Based on the chosen *ED*, children were assumed to use all the product categories. Hands were assumed as the only route of dermal contact. Exposed children were divided into two groups by age (i.e., 0–2 years and 3–12 years) for time-effective simulation and by considering different physiological and behavioral (mouthing) patterns [27]. For other parameters, except *MR*, *CA*, *D*, *ED*, *C_art_*, *SA,* and *BW*, the same default values in CEM were used as in Section 2.4.2.

## 3. Results

### 3.1. Occurrence of Phthalate Esters and DEHA

**Figure 1:**
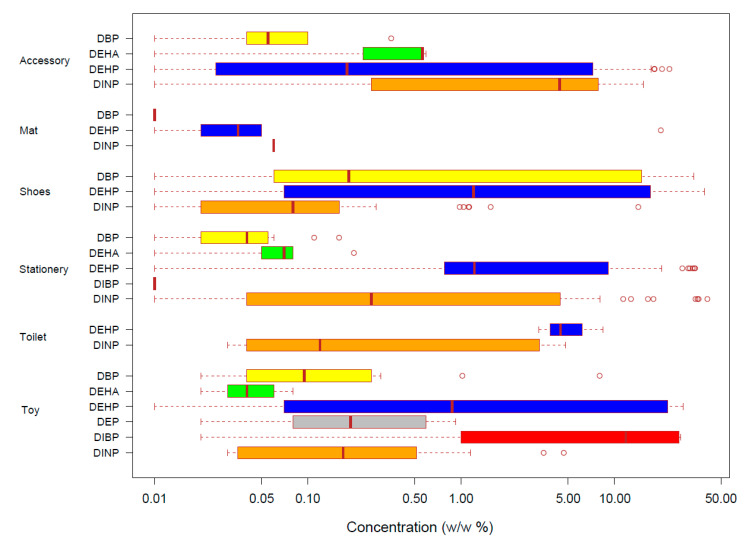
Range of plasticizers’ concentration in six categories of children’s products in which their concentration was above the detection limits. The box plot describes the minimum, 25 percentile, median, 75 percentile, and the maximum values, with outliers as open circles. Each chemical is expressed in different colors (DBP: yellow, DEHA: green, DEHP: blue, DEP: gray, DIBP: red, and DINP: orange).

Among the analyzed 3345 products, at least one plasticizer was determined above the detection limit in 387 products, and only one plasticizer was determined in 286 products. DEP was the only chemical used singularly in 10 toys, but DEHP was found in all samples containing more than 1 plasticizer with the exceptions of two products in shoes category in which the combination of DINP and DBP was found. The most frequently detected plasticizer was DEHP in 264 products, followed by DINP (n = 141), DBP (n = 66), DEHA (n = 30), DIBP (n = 14), and DEP (n = 10). DEHP was found in all product categories. Figure 1 presents the range of plasticizer contents in six product categories in which each plasticizer was detected. In accessories, the highest concentrations of DBP, DEHA, DEHP, and DINP were 0.35, 0.59, 22.90, and 15.52% (*w*/*w*), respectively. In mat, the highest concentrations of DBP, DEHP, and DINP were 0.01, 20.13, and 0.06% (*w*/*w*), respectively. In shoes, the highest concentrations of DBP, DEHP, and DINP were 33.12, 38.95, and 14.34% (*w*/*w*), respectively. In stationery, the highest concentrations of DBP, DEHA, DEHP, DIBP, and DINP were 0.16, 0.20, 33.68, 0.01, and 40.52% (*w*/*w*), respectively. In toilet, the highest concentrations of DEHP and DINP were 8.41, and 4.83% (*w*/*w*), respectively. In toy, the highest concentrations of DBP, DEHA, DEHP, DEP, DIBP, and DINP were 8.03, 0.08, 28.11, 0.92, 27.20, and 4.68% (*w*/*w*), respectively. The highest concentration of plasticizer in analyzed products was 40.52% (*w*/*w*) of DINP in an eraser in the stationery category. The concentrations of DEHP were relatively high in all categories but not always the highest. The high detection frequency and concentration of DEHP might be associated with its good performance as a plasticizer and relatively low price [3,19].

### 3.2. Estimated Exposure and Margin of Exposure (MOE)

The exposure ranges of phthalate plasticizers in children’s products were estimated by both deterministic and probabilistic estimation. Table 3 summarizes the values of the reference dose, calculated exposure ranges between the maximum and minimum, and the MOE values by product category. The exposure ranges by ingestion were estimated only for accessories, stationery, and toy categories, because it is unlikely that oral ingestion is significant for other categories. The maximum and minimum exposure limits were estimated between 5.0 × 10^−1^ and 5.0 × 10^−3^ mg kg^−1^ d^−1^. Two discrete values of the migration rates, 10 and 0.1 mg cm^−2^ h^−1^, were assigned at the concentration of chemical greater than and less than 1% (*w*/*w*). The oral exposure to DEHP and DINP by three categories was 5.0 × 10^−1^ mg kg^−1^ d^−1^, because the highest concentration of DEHP and DINP exceeded 1%. The exposure to DBP by accessories and stationery was 5.0 × 10^−3^ mg kg^−1^ d^−1^; in the case of toy, the value was 5.0 × 10^−1^ mg kg^−1^ d^−1^, where the concentrations of two products (dart and doll) were higher than 1%. DEHA and DEP had only 5.0 × 10^−3^ mg kg^−1^ d^−1^ in all categories because of their low concentrations. The dermal exposure ranged from 4.9 × 10^−7^ to 1.8 mg kg^−1^ d^−1^ in all categories. The maximum exposure to DEHP via dermal absorption was higher because of its high contents. The MOE values based on the reference doses ranged over seven orders of magnitude, 1.1 to 3.1 × 10^7^. The highest MOEs were observed for DINP, because the dermal absorption is the only exposure pathway in categories of mat, shoes, and toilet.

Table 4 presents the exposure ranges estimated by the probabilistic approach and its corresponding MOE values. Exposure through ingestion is estimated to be the highest in DEHP at 0.416 mg kg^−1^ d^−1^. The estimated exposure through ingestion in the same age group differs by chemicals within two orders of magnitude difference. The higher exposure in the younger age group to all plasticizers through ingestion is attributed to the higher frequency in mouthing behavior. For the dermal route, the exposure to DBP is the highest, 0.157 mg kg^−1^ d^−1^ (95th percentile in 3–12 years group), followed by DEHP and DEP. Unlike ingestion, differences between age groups were not as significant through dermal contacts. As exposure by ingestion is estimated to be 10 to 1000 times greater than by dermal contact, ingestion of the products determined the total exposure. Based on the total estimated exposure, the 95th percentile MOE values in all age groups were less than 100 [34,35] in DBP and DEBP. The MOE value was the highest in DEP for the younger age group with 49,000 (95th percentile in 0–2 years group), followed by DEHA and DIBP.

## 4. Discussion

The level of phthalate plasticizers in children’s products was similar to those reported in other studies conducted in recent decades [12,13,14,15,16,17,18]. The optimal amount of phthalate plasticizers to be added in PVC and other plastics is likely to depend on the purpose of their addition, unless they are unintentionally added during production processes. There were no noticeable temporal trends in the patterns of plasticizer use in all analyzed products purchased within three years. Nevertheless, DEHP and DINP are the two most predominant phthalate plasticizers. The concentration of DEHP is the highest in most products in all categories. DBP and DEP were found steadily in children’s products, although the detection frequency is low and the amount in products was minor. DIBP was detected in several products in the toy category with relatively high concentrations. Recently, DEHA has started appearing in products, as it is an alternative to DEHP [36,37].

The estimated MOE values in this study are relatively low, although the exposure was calculated by the single product category or chemical. Estimated total exposure using deterministic and probabilistic ways in this study is slightly greater than those in previous literature [38,39,40,41,42]. Lioy et al. reported the estimated daily phthalate intake from children’s products and comparing with urine monitoring from several studies [40]. In the case of children, exposure through diet was the dominant pathway explaining more than 40% of total exposure to DEHP, followed by the use of childcare products (>20%) and toys (>10%). Kim et al. analyzed phthalate metabolites in the urine of elementary school children of Korea [41]. The converted daily exposure to DEHP was comparable with the result of DEHP in 3–12 years old age group in this study. Those bio-monitored exposure ranges are similar to the results of this study. As the daily intake estimated in the previous literature [40,41,42] includes all possible exposure routes including foods and dust, the exposure estimation in this study could be greater than those by the reverse-dosimetric estimation from biomonitoring. Dermal exposure estimated in this study shows similar ranges with the total exposure from those references, suggesting that the estimated level is rather high, because the daily intakes from the literature include both dietary and non-dietary routes.

The estimated exposure by a mathematical model is higher than that by human biomonitoring owing to the setting of exposure parameters preventing underestimation [22,35,39]. Likewise, the exposure algorithms adopted from CEM might exaggerate the exposure to plasticizers in children’s products. The calculation of daily dose from ingestion requires the migration rate in the digestive tracts. The migration rate can be affected by many variables such as temperature, surrounding matrix, and migration time [43,44]. In CEM, however, the migration rate values are assigned to four discrete values, 10, 0.1, 1 × 10^−3^, and 1 × 10^−4^ mg cm^−2^ h^−1^, based on the concentration in products. Because the concentration of plasticizers in products was often very close to 1% (10,000 ppm), a huge deviation (100-fold) in the migration rate resulted from slight differences in plasticizer contents. Moreover, the suggested migration rates in CEM could be much higher than that measured in our study using in vivo and in vitro systems [33]. Various analytic methods (e.g., chewing or sucking as in vivo methods and shaking, tumbling, or ultrasonication as in vitro methods) were used to measure the migration rate. The reported migration rates of DINP ranged from 1.0 × 10^−4^ to 8.3 × 10^−2^ mg cm^−2^ h^−1^ for children’s products [33]. Furthermore, all concentrations of DINP in reported products were higher than 1%; thus, the suggested migration rate would be 10 mg cm^−2^ h^−1^ according to the CEM’s exposure algorithm. Three to five orders of magnitude greater values of the migration rate than experimental values lead to the very low MOEs in this screening-level risk assessment based on the content of plasticizers. For a refined assessment, there is a need to obtain a reasonable migration rate using experimental determination or by a refined model.

In the case of daily dose from dermal absorption, the estimated exposure strongly depends on *FA*, although dermal exposure did not contribute to the overall exposure in this study. Low *FA* for DEHA, DEHP, and DINP owing to low water solubility resulted in lower dermal exposure [45]. Estimation of *FA* based on water solubility could be an important source of uncertainty, because there are significant uncertainties in water solubility of sparingly soluble chemicals. In addition, *FA* also depends on the chemical’s solubility in skin surface lipids [44]. The migrated chemicals from products could be absorbed through skin surface matrices consisting of water and lipid; the dominant pathway depends on their affinity with water or lipid.

## 5. Conclusions

In this study, the concentrations of six plasticizers were analyzed in 3345 children’s products purchased from 2017 to 2019 in Korea. Among them, 387 products contained at least one plasticizer over the detection limit, indicating that the use of phthalate plasticizers remains common in children’s products. DEHP and DINP were frequently found in all categories. There is no significant temporal trend of phthalate use, due to the relatively short sampling duration.

The exposure to phthalate plasticizers via oral ingestion and dermal absorption and the corresponding MOEs were estimated by deterministic and probabilistic estimation. Overall, our results were relatively higher than those in the literature. Possible overestimation by exposure models is likely due to uncertainties in key parameters. The migration rate should be refined more precisely in the oral ingestion pathway, and the absorbed fraction, *FA*, should be a key parameter for estimating dermal exposure.

## Figures and Tables

**Table 1:** Classification of purchased products and number of chemicals detected.

Category	Number of Products	Sub-Category of Products (Total Number of Samples)		Number of Products in Which Chemicals Were Above Detection Limits	
				Chemical	Number
Accessories	605	Clothes (116), jewelry (240), mobile accessories (85), household stuffs (82), DIY tools (82)		DBP	6
				DEHA	15
				DEHP	51
				DINP	29
Mat	110	Non-slip mat (11), play mat (99)		DBP	1
				DEHP	6
				DINP	1
Shoes	176	Roller shoes (25), sandals (36), summer shoes (59), indoor shoes (42), shower sandals (14)		DBP	36
				DEHP	30
				DINP	29
Stationery	785	Beauty and personal care (10), kitchen stuffs (41), office products (552), painting and drawing supplies (82), tools and furniture (100)		DBP	11
				DEHA	6
				DEHP	126
				DIBP	1
				DINP	54
Toilet	38	Potty toilet (38)		DEHP	5
				DINP	9
Toy	1631	Gift and party goods (168), play figures (780), shape and size of certain toys (75), arts and crafts (537), preschool Games (11), aquatic toys (37), other infant toys (23)		DBP	12
				DEHA	9
				DEHP	46
				DEP	10
				DIBP	13
				DINP	19
Sum	3345	-			

DBP (dibutyl phthalate), DEHA (di(e-ethylhexyl)adipate), DEHP (di(2-ethylhexyl)phthalate), DEP (diethyl phthalate), DIBP (di-isobutyl phthalate), DINP (diisononyl phthalate).

**Table 2:** Input parameters for the estimation of exposure.

Parameter	Description [Units]
*a*	Constant [-]
*AT*	Averaging time [d]
*BW*	Body weight [kg]
*CA*	Contact area of mouthing [cm^2^]
*C_art_*	Chemical concentration in article [mg cm^−3^]
*x*	Ratio of the evaporation rate from the SC surface to the dermal absorption rate through the SC [-]
*D*	Solid phase diffusion coefficient [m^2^ h^−1^]
*D_mouthing_*	Duration of mouthing [min h^−1^]
*Dur*	Duration of article contact [min]
*ED*	Exposure duration [d]
*FA*	Fraction absorbed [-]
*h*	Gas phase mass transfer coefficient [m h^−1^]
*h_sc_*	Stratum corneum (SC) thickness (assumed to be 15 μm)
*K_p_*	Permeability coefficient for chemical transport through the SC from an aqueous vehicle [cm h^−1^]
*l*	Average distance a diffusing molecule travels per contact [cm]
*MR*	Migration rate of chemical from article to saliva [mg cm^−2^ h^−1^]
*MW*	Molecular weight [mg mmol^−1^]
*p_vap_*	Vapor pressure [Torr]
*R*	Real gas constant [62.37 mL Torr K^−1^ mmol^−1^)
*SA*/*BW*	Surface area to body weight ratio [cm^2^ kg^−1^]
*S_w_*	Water solubility [mg mL^−1^]
*T*	Temperature [K]
*t_lag_*	Lag time for chemical transport through the SC [h]

**Table 3:** Estimated the maximum and minimum exposure ranges and calculated margin of exposure (MOE) from reference dose.

Category	Chemical	Reference Dose (mg kg^−1^ d^−1^)	Exposure Range (mg kg^−1^ d^−1^)						MOE	
			Ingestion		Dermal		Total			
			Max	Min	Max	Min	Max	Min	Max	Min
Accessories	DBP	2 ^1^	5.0 × 10^−3^	5.0 × 10^−3^	1.9 × 10^−2^	5.5 × 10^−4^	2.4 × 10^−2^	5.5 × 10^−3^	8.2 × 10^1^	3.6 × 10^2^
	DEHA	170 ^2^	5.0 × 10^−3^	5.0 × 10^−3^	2.0 × 10^−4^	3.5 × 10^−6^	5.2 × 10^−3^	5.0 × 10^−3^	3.3 × 10^4^	3.4 × 10^4^
	DEHP	3 ^3^	5.0 × 10^−1^	5.0 × 10^−3^	2.0 × 10^−2^	8.6 × 10^−6^	5.1 × 10^−1^	5.0 × 10^−3^	5.8 × 10^0^	6.1 × 10^2^
	DINP	15 ^6^	5.0 × 10^−1^	5.0 × 10^−3^	7.6 × 10^−4^	4.9 × 10^−7^	5.0 × 10^−1^	5.0 × 10^−3^	3.0 × 10^1^	3.0 × 10^3^
Mat	DBP	2 ^1^			5.5 × 10^−4^	5.5 × 10^−4^	5.5 × 10^−4^	5.5 × 10^−4^	3.6 × 10^3^	3.6 × 10^3^
	DEHP	3 ^3^			1.7 × 10^−2^	8.6 × 10^−6^	1.7 × 10^−2^	8.6 × 10^−6^	1.7 × 10^2^	3.5 × 10^5^
	DINP	15 ^6^			2.9 × 10^−6^	2.9 × 10^−6^	2.9 × 10^−6^	2.9 × 10^−6^	5.1 × 10^6^	5.1 × 10^6^
Shoes	DBP	2 ^1^			1.8 × 10^0^	5.5 × 10^−4^	1.8 × 10^0^	5.5 × 10^−4^	1.1 × 10^0^	3.6 × 10^3^
	DEHP	3 ^3^			3.4 × 10^−2^	8.6 × 10^−6^	3.4 × 10^−2^	8.6 × 10^−6^	8.9 × 10^1^	3.5 × 10^5^
	DINP	15 ^6^			7.0 × 10^−4^	4.9 × 10^−7^	7.0 × 10^−4^	4.9 × 10^−7^	2.1 × 10^4^	3.1 × 10^7^
Stationery	DBP	2 ^1^	5.0 × 10^−3^	5.0 × 10^−3^	8.8 × 10^−3^	5.5 × 10^−4^	1.4 × 10^−2^	5.5 × 10^−3^	1.5 × 10^2^	3.6 × 10^2^
	DEHA	170 ^2^	5.0 × 10^−3^	5.0 × 10^−3^	6.9 × 10^−5^	3.5 × 10^−6^	5.0 × 10^−3^	5.0 × 10^−3^	3.4 × 10^4^	3.4 × 10^4^
	DEHP	3 ^3^	5.0 × 10^−1^	5.0 × 10^−3^	2.9 × 10^−2^	8.6 × 10^−6^	5.2 × 10^−1^	5.0 × 10^−3^	5.7 × 10^0^	6.1 × 10^2^
	DIBP	125 ^5^	5.0 × 10^−3^	5.0 × 10^−3^	1.2 × 10^−5^	1.2 × 10^−5^	5.0 × 10^−1^	5.0 × 10^−1^	2.5 × 10^2^	2.5 × 10^2^
	DINP	15 ^6^	5.0 × 10^−1^	5.0 × 10^−3^	2.0 × 10^−3^	4.9 × 10^−7^	5.0 × 10^−1^	5.0 × 10^−3^	3.0 × 10^1^	3.0 × 10^3^
Toilet	DEHP	3 ^3^			7.3 × 10^−3^	2.8 × 10^−3^	7.3 × 10^−3^	2.8 × 10^−3^	4.1 × 10^2^	1.1 × 10^3^
	DINP	15 ^6^			2.4 × 10^−4^	1.5 × 10^−6^	2.4 × 10^−4^	1.5 × 10^−6^	6.3 × 10^4^	1.0 × 10^7^
Toy	DBP	2 ^1^	5.0 × 10^−1^	5.0 × 10^−3^	4.4 × 10^−1^	1.1 × 10^−3^	9.4 × 10^−1^	6.0 × 10^−3^	2.1 × 10^0^	3.3 × 10^2^
	DEHA	170 ^2^	5.0 × 10^−3^	5.0 × 10^−3^	2.8 × 10^−5^	6.9 × 10^−6^	5.0 × 10^−3^	5.0 × 10^−3^	3.4 × 10^4^	3.4 × 10^4^
	DEHP	3 ^3^	5.0 × 10^−1^	5.0 × 10^−3^	2.4 × 10^−2^	8.6 × 10^−6^	5.2 × 10^−1^	5.0 × 10^−3^	5.8 × 10^0^	6.1 × 10^2^
	DEP	750 ^4^	5.0 × 10^−3^	5.0 × 10^−3^	8.7 × 10^−2^	1.9 × 10^−3^	9.2 × 10^−2^	6.8 × 10^−3^	8.2 × 10^3^	1.1 × 10^5^
	DIBP	125 ^5^	5.0 × 10^−1^	5.0 × 10^−3^	3.3 × 10^−2^	2.5 × 10^−5^	5.3 × 10^−1^	5.0 × 10^−3^	2.4 × 10^2^	2.5 × 10^4^
	DINP	15 ^6^	5.0 × 10^−1^	5.0 × 10^−3^	2.3 × 10^−4^	1.5 × 10^−6^	5.0 × 10^−1^	5.0 × 10^−3^	3.0 × 10^1^	3.0 × 10^3^

^1^ LOAEL, endpoint: germ cell development, species: rat [28], ^2^ NOAEL, endpoint: changes in bodyweight and liver weight, species: rat [29], ^3^ NOAEL, endpoint: nipple retention, species: rat [30], ^4^ NOAEL, endpoint: decreased growth rate, species: rat [31] ^5^ NOAEL, endpoint: nipple retention, species: rat [32], ^6^ NOAEL, endpoint: hepatotoxicity, species: rat [33], DBP: dibutyl phthalate; DEHA: di(e-ethylhexyl)adipate); DEHP: di(2-ethylhexyl)phthalate); DEP: diethyl phthalate; DIBP: di-isobutyl phthalate; DINP: diisononyl phthalate.

**Table 4:** Estimated exposure ranges via ingestion and dermal contact of chemicals in children’s products and their corresponding margin of exposure (MOE) from reference doses

Chemical	Age Group	Exposure Range (mg kg^−1^ d^−1^)						MOE	
		Ingestion		Dermal		Total			
		Median	95%	Median	95%	Median	95%	Median	95%
DBP	0–2 years	1.5 × 10^−2^	9.6 × 10^−2^	8.2 × 10^−3^	1.2 × 10^−1^	3.2 × 10^−2^	1.9 × 10^−1^	6.2 × 10^1^	1.1 × 10^1^
	3–12 years	2.2 × 10^−3^	1.5 × 10^−2^	8.9 × 10^−3^	1.6 × 10^−1^	1.4 × 10^−2^	1.7 × 10^−1^	1.5 × 10^2^	1.2 × 10^1^
DEHA	0–2 years	1.6 × 10^−2^	9.1 × 10^−2^	1.9 × 10^−6^	1.8 × 10^−5^	1.6 × 10^−2^	9.1 × 10^−2^	1.1 × 10^4^	1.9 × 10^3^
	3–12 years	2.3 × 10^−3^	1.5 × 10^−2^	9.1 × 10^−7^	9.8 × 10^−6^	2.3 × 10^−3^	1.5 × 10^−2^	7.5 × 10^4^	1.2 × 10^4^
DEHP	0–2 years	2.0 × 10^−2^	4.2 × 10^−1^	3.0 × 10^−4^	3.0 × 10^−3^	2.1 × 10^−2^	4.2 × 10^−1^	1.4 × 10^2^	7.2 × 10^0^
	3–12 years	3.8 × 10^−3^	7.2 × 10^−2^	2.2 × 10^−4^	2.9 × 10^−3^	4.7 × 10^−3^	7.5 × 10^−2^	6.4 × 10^2^	4.0 × 10^1^
DEP	0–2 years	1.6 × 10^−2^	9.1 × 10^−2^	5.6 × 10^−4^	1.3 × 10^−3^	1.6 × 10^−2^	9.2 × 10^−2^	2.9 × 10^5^	4.9 × 10^4^
	3–12 years	2.3 × 10^−3^	1.5 × 10^−2^	2.6 × 10^−4^	1.0 × 10^−3^	2.6 × 10^−3^	1.5 × 10^−2^	4.8 × 10^4^	7.4 × 10^3^
DIBP	0–2 years	1.5 × 10^−2^	1.0 × 10^−1^	9.4 × 10^−6^	8.1 × 10^−5^	1.6 × 10^−2^	1.0 × 10^−1^	8.1 × 10^3^	1.2 × 10^3^
	3–12 years	2.3 × 10^−3^	1.5 × 10^−2^	3.7 × 10^−6^	1.9 × 10^−5^	2.3 × 10^−3^	1.5 × 10^−2^	5.5 × 10^4^	8.3 × 10^3^
DINP	0–2 years	1.7 × 10^−2^	3.8 × 10^−1^	3.1 × 10^−6^	4.4 × 10^−5^	1.7 × 10^−2^	3.8 × 10^−1^	8.8 × 10^2^	3.9 × 10^1^
	3–12 years	2.6 × 10^−3^	6.7 × 10^−2^	2.1 × 10^−6^	3.3 × 10^−5^	2.6 × 10^−3^	6.7 × 10^−2^	5.8 × 10^3^	2.2 × 10^2^

DBP: dibutyl phthalate; DEHA: di(e-ethylhexyl)adipate); DEHP: di(2-ethylhexyl)phthalate); DEP: diethyl phthalate; DIBP: di-isobutyl phthalate; DINP: diisononyl phthalate
